# Effectiveness of Traditional Chinese Fitness Methods on Depressive Symptoms and Glycaemic Control in Patients With Type 2 Diabetes and Comorbid Depression: A Systematic Review and Meta‐Analysis of Taijiquan and Baduanjin

**DOI:** 10.1002/edm2.70356

**Published:** 2026-11-01

**Authors:** Xinyue Tian, Yiwen Wei, Jingwen Bo, Keke Lin, Yina Shen, Yu Liu

**Affiliations:** ^1^ School of Nursing Beijing University of Chinese Medicine Beijing People's Republic of China; ^2^ Department of Nursing China‐Japan Friendship Hospital Beijing People's Republic of China

**Keywords:** Baduanjin, depression, fitness, glycaemic control, Taijiquan, traditional Chinese medicine, Type 2 diabetes mellitus

## Abstract

**Aims:**

To evaluate the overall effectiveness of traditional Chinese fitness methods (TCFMs)—specifically Taijiquan and Baduanjin—on depressive symptoms and glycaemic control in patients with Type 2 diabetes (T2DM) and comorbid depression.

**Methods:**

RCTs evaluating the effectiveness of TCFMs were identified through 10 major English and Chinese databases, including PubMed, EMBASE, Cochrane Central Register of Controlled Trials, Web of Science, Wiley Online Library, ProQuest, CBM, CNKI, VIP, and Wanfang Data. Risk of bias was assessed using the revised Cochrane RoB 2 tool, and evidence certainty was graded using GRADE. Meta‐analyses were conducted using the DerSimonian‐Laird random‐effects model. Subgroup analyses were performed by intervention duration, frequency, and sample size.

**Results:**

Eight RCTs of Taijiquan and Baduanjin were included. All studies were conducted in China, and most had small sample sizes. Most had a high risk of bias, mainly due to a lack of blinding of participants and personnel. TCFMs were associated with significant improvements in depressive symptoms (SMD = −1.39, 95% CI [−2.22, −0.57], *p* = 0.001). Significant improvements were also observed for HbA1c (MD = −0.82, 95% CI [−1.18, −0.45], *p* < 0.0001) and FPG (MD = −0.70, 95% CI [−0.97, −0.42], *p* < 0.00001). The effect on 2hPBG remains inconclusive owing to limited studies. Subgroup analyses revealed no significant effect modifiers for depressive symptoms or HbA1c (all interaction *p* > 0.05). Sensitivity analyses confirmed the robustness of the primary findings, though heterogeneity decreased substantially after excluding high‐risk studies.

**Conclusions:**

This review provides preliminary evidence that TCFMs may confer benefits on mental health and glycaemic regulation in patients with T2DM and comorbid depression. However, given the low certainty of the evidence, high heterogeneity, and methodological limitations, these findings should be interpreted cautiously. The conclusions are preliminary and require confirmation in larger, rigorously designed studies.

**Trial Registration:**

The protocol of this systematic review was registered on PROSPERO (CRD420261287519)

## Graphical abstract

Traditional Chinese fitness methods show preliminary benefits for depression and glycaemic control in patients with Type 2 diabetes and comorbid depression; optimal regimens are unconfirmed due to limited evidence.

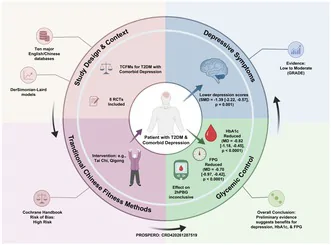

## 1 Introduction

In recent years, with population ageing and lifestyle changes, diabetes prevalence in China has continued to rise. Among adults in China, the prevalence reaches 11.2%, with Type 2 diabetes mellitus (T2DM) accounting for over 90% of all cases [1]. Long‐term strict diet and pharmacological treatment increase psychological stress, predisposing T2DM patients to depression [2]. Globally, the prevalence of depression in patients with T2DM ranges from approximately 10% to 30%, roughly twice that observed in people without diabetes [3, 4]. Depression can disrupt diabetes self‐management behaviours, thereby accelerating disease progression and contributing to poorer prognosis. Diabetes and depression share a bidirectional relationship, reinforcing each other through neuroendocrine dysregulation, chronic inflammation, and lifestyle factors—a self‐perpetuating metabolic–psychological cycle [5]. In addition to pharmacological and psychological therapies, exercise may also help alleviate depressive symptoms in patients with T2DM and comorbid depression while exerting beneficial effects on glycaemic control [6].

Among exercise interventions, Traditional Chinese Fitness Methods (TCFMs), which originate from traditional Chinese health cultivation practices, have attracted increasing attention in recent years. TCFMs are mind–body practices that integrate slow, controlled movements with breath regulation and meditative attention. Although various forms exist—including Taiji (e.g., Taijiquan) and Qigong (e.g., Baduanjin)—they share core principles of regulating the body, breath, and mind, with an emphasis on Qi circulation and Yin‐Yang balance [7, 8]. TCFMs may yield synergistic benefits for both glycaemic control and psychological well‐being [9, 10]. Accordingly, international systematic reviews often classify “mind–body exercise” as an independent category, commonly grouping Taiji, Qigong, yoga, and related practices, with TCFMs regarded as an important subset within this broader domain [11, 12]. However, while these traditional concepts provide a culturally grounded framework for understanding TCFMs, they remain largely theoretical and hypothesis‐generating. From a biomedical perspective, the benefits of TCFMs may be explained through neuropsychological mechanisms (e.g., dopamine/endorphin release and cortisol reduction) and metabolic pathways (e.g., improved glucose metabolism and insulin sensitivity) [13, 14, 15, 16]. Preliminary evidence suggests that Taijiquan and Baduanjin may improve both glycaemic control and depressive symptoms in patients with T2DM and comorbid depression [17, 18, 19].

Previous systematic reviews have examined Taiji or Qigong for either glycaemic control or depressive symptoms in broader populations, but none has simultaneously evaluated both outcomes in patients with confirmed comorbid depression. In 2022, Kong et al. reviewed Baduanjin for depressive symptoms in T2DM [20], but systematic reviews treating TCFMs as a comprehensive category for this specific comorbid population remain scarce. Meanwhile, existing systematic reviews on Qigong or Taiji for glycaemic outcomes in this population have reached inconsistent conclusions: Some regard the impact on HbA1c as uncertain [21, 22], while others report favourable results [23, 24]. These gaps in the evidence base—namely, the absence of a synthesised evaluation of TCFMs for depression in this specific comorbid population, and the unresolved discrepancy regarding glycaemic benefits—necessitate a more rigorous and comprehensive synthesis. To address these issues, the present review differs from prior work in four key methodological respects: (1) simultaneously assessing depressive symptoms and glycaemic outcomes within a single framework, rather than examining them in isolation; (2) including studies up to January 2026 to incorporate the most current evidence; (3) restricting to patients with confirmed comorbid depression, allowing for a more targeted and clinically meaningful interpretation; and (4) conducting prespecified subgroup analyses to explore dose‐related factors—a dimension largely overlooked in previous reviews.

This systematic review and meta‐analysis aims to provide a comprehensive and reliable assessment of the combined effects of TCFMs on depressive symptoms and glycaemic outcomes in patients with T2DM and comorbid depression.

## 2 Methods

This study was a systematic review and meta‐analysis of randomised controlled trials (RCTs), following the methodological specifications of the Cochrane Handbook for the Systematic Review of Interventions (Version 6.5) [25]. The aim was to synthesise evidence on the effectiveness of TCFMs in improving depression and blood glucose indices of patients with T2DM and comorbid depression, and to analyse the similarities and differences among different methods through subgroup analysis. The protocol was prospectively registered in the International Prospective Register of Systematic Reviews (PROSPERO, CRD420261287519).

### 2.1 Search Strategy

Two independent reviewers searched the following 10 electronic databases: Cochrane Central Register of Controlled Trials, PubMed, EMBASE, Wiley Online Library, Web of Science, ProQuest, China Biology Medicine Database (CBM), China National Knowledge Infrastructure (CNKI), VIP Journal Integration Platform, and Wan‐fang Data Knowledge Service Platform (Wan‐Fang database) from their inception to 3 January 2026.

A systematic literature search was conducted using subject headings (MeSH) combined with a free‐text term search. Studies in Chinese from the inception to January 2026 available on CBM, CNKI, Wan‐Fang database, and VIP Journal Integration Platform were searched using the keywords (in Chinese): “Diabetes mellitus”, “Xiaoke”, “Depression”, “Qigong”, “Taiji”, “Baduanjin”, “Wuqinxi”, “Liuzijue”, “Yijinjing”, “Shierduanjin”, “Dawu”, “Daoyin Yangsheng Gong 12‐movement”, “Mawangdui Daoyin”, “Jingsong Gong”, “Fangsong Gong”, and “Zhanzhuang Gong”. Research studies in English from the inception to January 2026 available on Cochrane Central Register of Controlled Trials, PubMed, EMBASE, Wiley Online Library, Web of Science, and ProQuest were searched using the keywords (in English), with the research strategy (using the PubMed search as an example) presented in Figure 1. The full search strategies for all 10 databases are provided in Table S1. The PRISMA 2020 checklist is provided as File S1. To partially mitigate the absence of systematic grey literature retrieval, we included dissertations identified through CNKI, Wanfang, and VIP. We did not systematically search conference proceedings, preprints, or unpublished trial registries, as preliminary scoping searches of these sources (e.g., clinicaltrials.gov, Chinese Clinical Trial Registry) identified very few potentially eligible records, and none met our full eligibility criteria upon review. We restricted the search to English and Chinese languages because TCFMs are predominantly studied and reported in these two language contexts, and preliminary searches identified no eligible studies in other languages.

**FIGURE 1:**
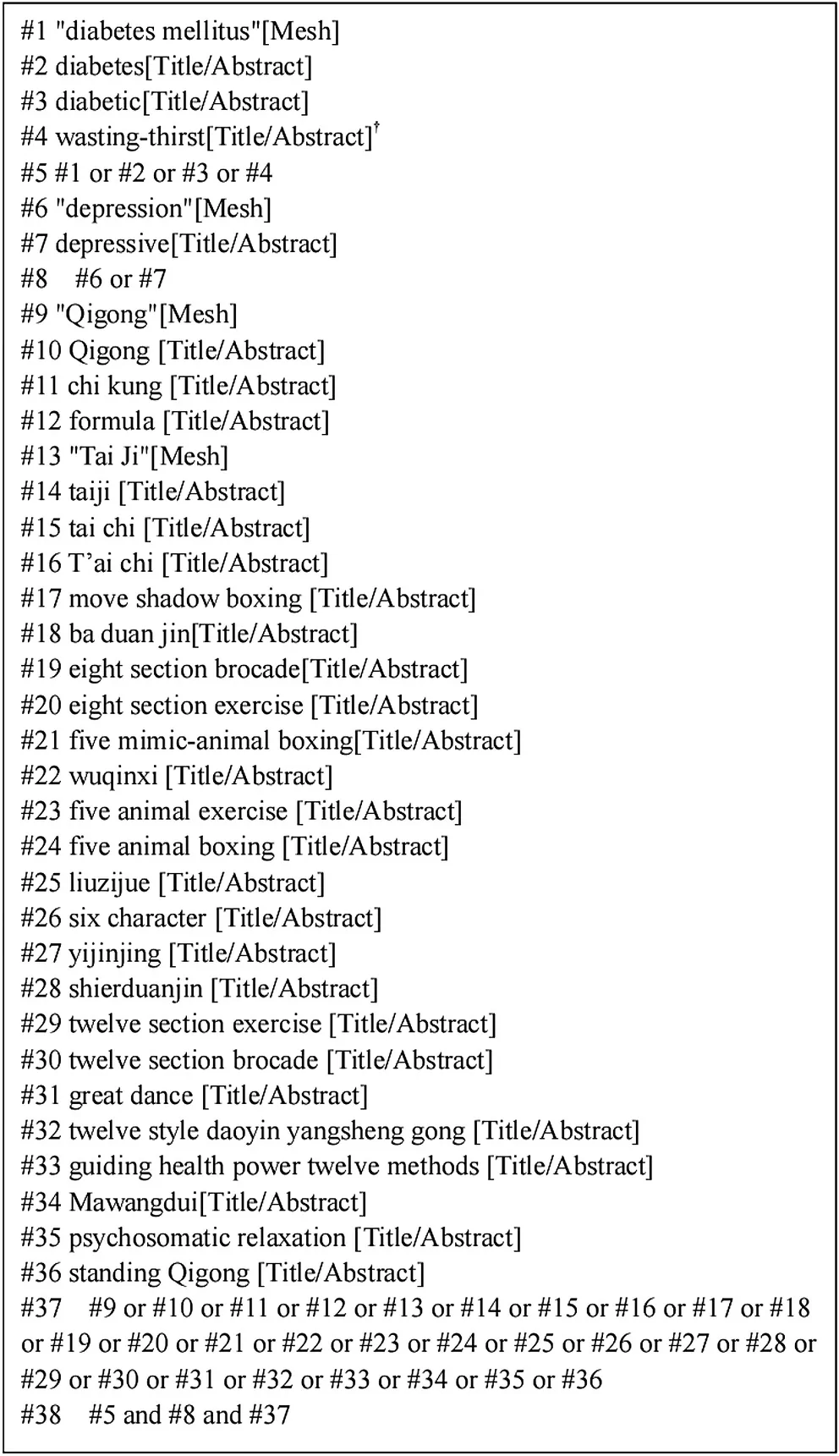
PubMed search strategy. († wasting‐thirst: Name of diabetes in traditional Chinese medicine).

### 2.2 Eligibility Criteria

Two independent reviewers screened abstracts using the following selection criteria: ① Research type: Randomised controlled trial (RCT). ② Participants: Patients with T2DM who had comorbid depression, as defined by validated screening instruments with specified cut‐off scores. ③ Intervention group and control group: Intervention groups received one or more Traditional Chinese Fitness Methods (TCFMs)—defined as mind–body practices originating from traditional Chinese health cultivation, including Taijiquan, Baduanjin, Wuqinxi, Liuzijue, and related forms—either alone or in conjunction with other interventions that were also received by the control group. Control groups received the same co‐interventions (e.g., conventional treatment, health education, or drug therapy) as the intervention groups, but did not receive TCFMs or any other form of exercise. Although the search strategy covered a broad range of TCFMs, only Taijiquan and Baduanjin were represented in the studies that met all eligibility criteria; these two forms therefore constitute the interventions examined in this review. ④ Outcome: The main outcomes included depression rating scale scores; secondary outcomes included HbA1c, fasting plasma glucose (FPG), and 2‐h postprandial blood glucose (2hPBG). ⑤ Language: Chinese and English. Repeated publications or literature with incomplete data were excluded.

### 2.3 Data Extraction and Quality Appraisal

Two independent reviewers extracted data using standardised tools in JBI SUMARI, including authors, year, literature type, sample size, age, intervention details (type, form, duration, frequency, intensity), outcome measures (depression and blood glucose indices), and measurement tools. Disagreements between reviewers were resolved through discussion or third‐party arbitration. Outcome data were extracted at the end of the intervention period. Risk of bias was assessed independently using the revised Cochrane RoB 2 tool across five domains, with overall judgement classified as low risk, some concerns, or high risk. Evidence certainty was graded using the GRADE approach (high, moderate, low, or very low). When necessary, original authors were contacted as the first priority to obtain missing or additional data. For studies that reported outcome data as median and interquartile range instead of mean and standard deviation, the sample mean and standard deviation were estimated using the method described by Wan et al. (2014) [26] when raw data were not available from the authors.

### 2.4 Statistical Analysis

The meta‐analyses were conducted using Review Manager 5.4. ① Continuous variable data were evaluated with mean difference (MD) or standardised mean difference (SMD) and reported with 95% confidence intervals (CIs). If studies were basically the same in interventions and outcome measures, they were combined for meta‐analysis. To ensure statistical robustness, meta‐analysis was performed only when at least three studies were available for a given outcome. For outcomes with fewer than three eligible studies, results were summarised descriptively without pooling effect estimates. A random‐effects model (DerSimonian‐Laird) was used a priori for all meta‐analyses. For studies that reported depression outcomes using more than one rating scale, only the data from the primary scale specified in the original study were extracted for the main meta‐analysis to avoid unit‐of‐analysis error and sample inflation. ② The combined effect size and 95% CI were calculated, and the difference was statistically significant with *p* < 0.05. ③ Subgroup analyses were performed for outcomes with substantial heterogeneity (I^2^ ≥ 50%) according to key study‐level factors, including intervention duration, frequency, and sample size. To ensure statistical reliability, subgroup classifications were only considered when each subgroup contained at least three studies. Other potential effect modifiers, such as session length, intervention type, combined interventions, age, and baseline depression severity, were not examined due to insufficient studies in each category or limited baseline variability. ④ Meta‐regression: If 10 or more studies were available, a univariate meta‐regression was planned for continuous covariates using the restricted maximum likelihood (REML) method. If fewer than 10 studies were included, no meta‐regression would be performed. ⑤ Sensitivity analysis: The robustness of the pooled effect estimates was assessed using the leave‐one‐out approach, sequentially excluding each study to evaluate its influence on the overall results. For the primary outcome (depression rating scale scores), additional sensitivity analyses were prespecified to sequentially exclude: Unclear randomisation, dissertations, high risk of bias, combined interventions, extreme effect sizes. Given the small number of included studies (*n* = 8) and the substantial heterogeneity observed, we performed supplementary analyses using the Hartung‐Knapp‐Sidik‐Jonkman (HKSJ) method with the Sidik‐Jonkman estimator for τ^2^ to assess the robustness of the confidence intervals for the primary outcome to the choice of statistical model. ⑥ Identification of publication bias: If the number of studies was 10 or more, funnel plots and Egger's regression test were planned to assess potential publication bias. If fewer than 10 studies were included, no tests would be performed.

## 3 Results

### 3.1 Study Characteristics

Originally, 2505 potentially relevant articles were identified, and 243 studies may have met the inclusion criteria but were excluded according to the exclusion criteria after reading the full text. Finally, eight RCT studies were selected. All the articles were from Chinese research institutes, and five of them were from Chinese journals. The process of literature screening is shown in Figure 2.

**FIGURE 2:**
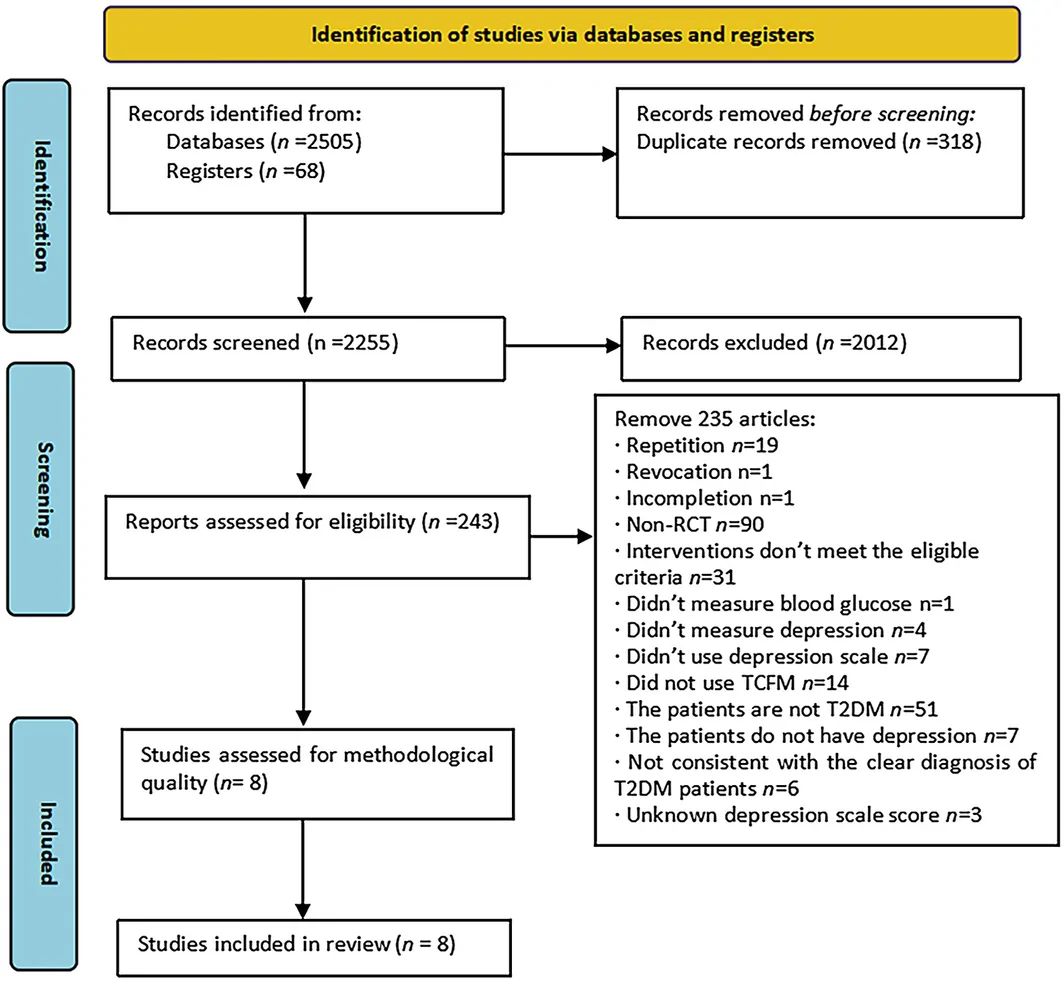
PRISMA flow diagram of search results and study selection and inclusion process.

### 3.2 Methodology Characteristics

The methodological characteristics of the included studies are shown in Table 1; the characteristics of TCFMs and the depression assessment tools are integrated in Table 2; and detailed baseline characteristics are provided in Table S2. Eight studies evaluated depressive symptoms using the Self‐rating Depression Scale (SDS), Hamilton Depression Scale (HAMD), Patient Health Questionnaire‐9 (PHQ‐9) or Montgomery‐Asberg Depression Rating Scale (MADRS). The sample size in the eight studies ranged from 20 to 105 cases, totalling 487.

**TABLE 1:** Methodology characteristics of the eight studies.

Research	Subject	Sample size (*n*) T/C	Age (year‐old)	Intervention		Intervention duration	Outcome index	Literature type
				T C				
Zhang 2011 [27]	T2DM and depression or anxiety	15/15	54.90 (mean)	Baduanjin + conventional treatment	Conventional treatment	6 months	①②③④	B
Liu 2012 [28]	T2DM and depression	33/36	64.20 ± 7.41	Baduanjin +community health education on T2DM	Community health education on T2DM	12 weeks	①②	A
Yang 2017 [29]	T2DM and depression	53/52	52.40 ± 2.30	Baduanjin + health education on T2DM	Health education on T2DM	12 weeks	①②	A
Li 2021 [30]	Middle‐aged patients with T2DM and depression	10/10	46.20 (mean)	24‐action Taijiquan + drug therapy	Drug therapy	14 weeks	①②③	B
Zu 2021 [31]	T2DM and anxiety and depression	34/34	55.65 (mean)	Modified Baduanjin + drug therapy	Drug therapy	4 weeks	①③	A
Song 2022 [32]	Elderly patients with T2DM and depression	10/10	65.70 (mean)	Baduanjin + drug therapy	Drug therapy	12 weeks	①②③	B
Han 2024 [33]	T2DM and moderate anxiety and depression	41/42	65.10 (mean)	Baduanjin + routine management on T2DM	Routine management on T2DM	6 months	①②③	A
Hu 2025 [34]	T2DM and depression	46/46	55.85 (mean)	24‐action Taijiquan + Shuganjieyu capsules + conventional treatment	Shuganjieyu capsules + conventional treatment	8 weeks	①②③④	A

*Note:* T‐Intervention group, C‐control group, A‐Journal article, B‐Master's & PhD thesis. ① depression scale score, ② HbA1c, ③ fasting plasma glucose (FPG), ④ 2 h postprandial blood glucose(2hPBG).

**TABLE 2:** Characteristics of interventions and depression assessment tools of the eight studies.

Research	Interventions						Depression assessment tools		
	Exercise type	Exercise form	Session length (min)	Frequency (time/week)	Duration	Standard for exercise intensity	Assessment tool	Diagnostic criteria	Baseline severity
Zhang 2011	Qigong	Baduanjin	60	7 (2 times massed practice +5 times distributed practice)	6 months	Not reported	MADRS	Scale‐based: MADRS ≥ 12 and item 10 < 2	Mild‐to‐moderate
Liu 2012	Qigong	Baduanjin	40	3 ~ 5	12 weeks	Not reported	SDS	Chinese norm: SDS standard score ≥ 53	Mild
Yang 2017	Qigong	Baduanjin	60	7 (1 time massed practice + 6 times distributed practice)	12 weeks	Not reported	HAMD‐24	Not explicitly stated; clinical assessment	Mild‐to‐moderate
Li 2021	Taiji	24‐action Taijiquan	60	7	14 weeks	100–110 time/cent	SDS	Scale‐based: SDS standard score ≥ 50	Mild
Zu 2021	Qigong	Modified Baduanjin	Not reported	7	4 weeks	Not reported	SDS	Scale‐based: SDS standard score ≥ 50	Mild
Song 2022	Qigong	Baduanjin	60	7	12 weeks	95–105 time/cent	SDS	Scale‐based: SDS standard score ≥ 50	Mild
Han 2024	Qigong	Baduanjin	60	5 (1 time massed practice + 4 times distributed practice)	6 months	Not reported	PHQ‐9	Scale‐based: PHQ‐9 5–14	Mild‐to‐moderate
Hu 2025	Taiji	24‐action Taijiquan	30–40	5	8 weeks	Not reported	HAMD‐17	CCMD‐3 criteria for depression	Moderate

Abbreviations: CCMD‐3, Chinese Classification of Mental Disorders, 3rd edition; HAMD, Hamilton Depression Scale; MADRS, Montgomery‐Asberg Depression Rating Scale; PHQ‐9, Patient Health Questionnaire‐9; SDS, Self‐Rating Depression Scale.

### 3.3 Risk of Bias

The risk of bias in the eight included studies was critically evaluated by two independent reviewers using the revised Cochrane risk‐of‐bias tool for randomised trials (RoB 2). As shown in Figures 3 and 4, for bias arising from the randomisation process, 3 studies [28, 32, 33] were judged as low risk, 4 studies as having some concerns, and 1 study [30] as high risk. For bias due to deviations from intended interventions, no study was rated low risk. Three studies [28, 30, 32] had some concerns. The remaining five studies were judged as high risk, mainly because blinding of participants and personnel was not feasible in exercise interventions.

**FIGURE 3:**
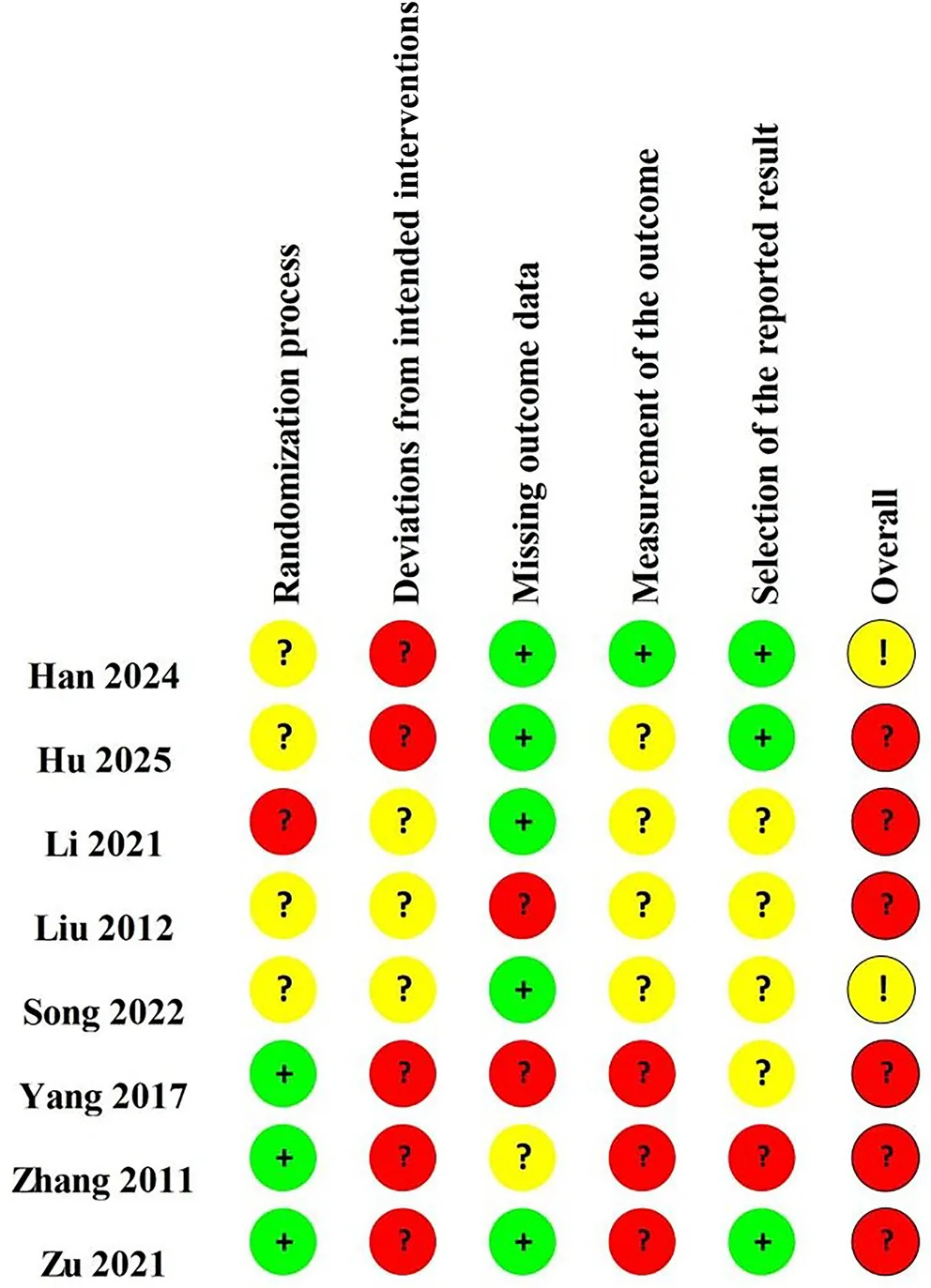
Risk‐of‐bias Summary Plot (RoB 2): Bias Judgements for Each of the 8 Included RCTs.

**FIGURE 4:**
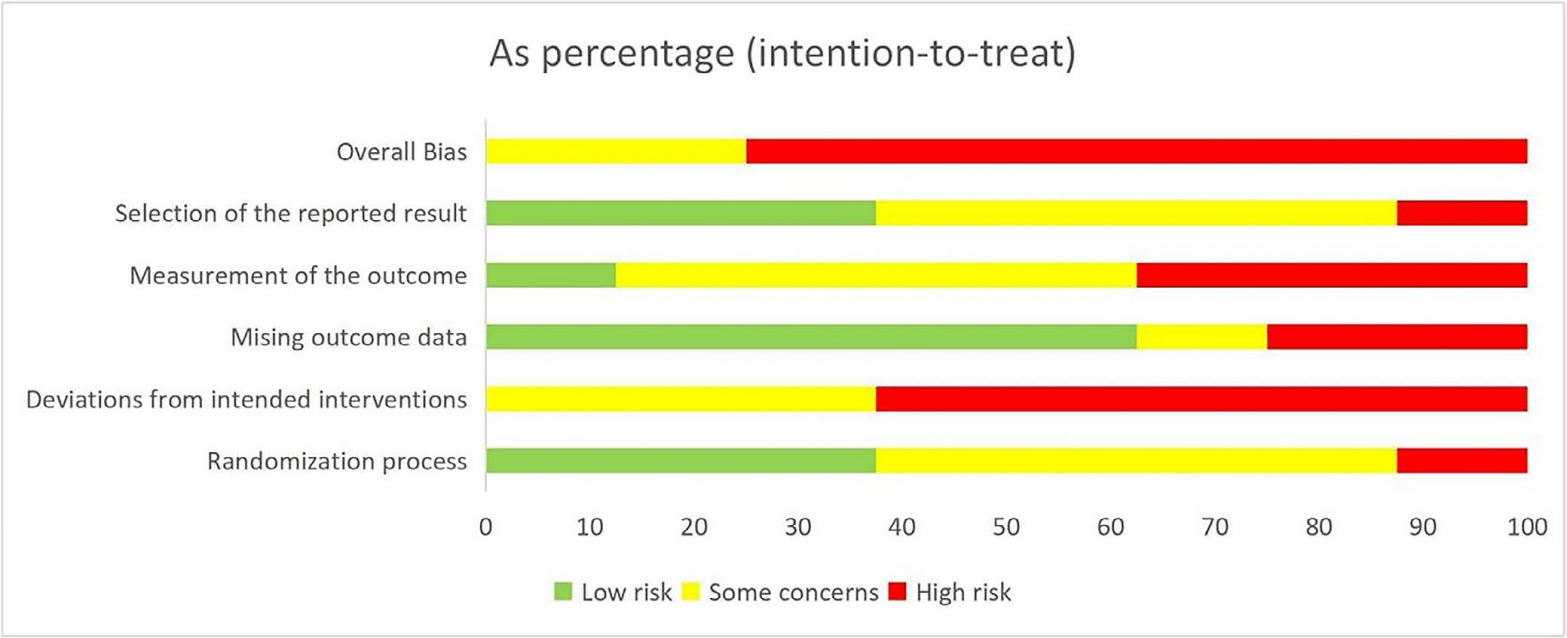
Risk‐of‐bias Distribution Bar Plot (RoB 2): Proportions of Judgements Across Bias Domains for the 8 Included RCTs.

For bias due to missing outcome data, 5 studies were low risk, 1 study [27] had some concerns, and 2 studies [28, 29] were high risk due to substantial and unbalanced dropout without appropriate correction. For bias in measurement of the outcome, 1 study [33] was rated low risk, 5 studies had some concerns, and 2 studies [29, 31] were high risk, largely because subjective scale assessments were not blinded to outcome assessors. For bias in selection of the reported result, 4 studies were low risk, 3 studies [29, 31, 34] had some concerns, and 1 study [27] was high risk due to the absence of a pre‐registered analysis plan and multiple eligible analyses. Consequently, the overall risk‐of‐bias judgement was high risk for 6 studies and some concerns for 2 studies [32, 33]; no study was judged as low risk overall. The predominance of high risk of bias was primarily attributable to the inherent difficulty of blinding participants and personnel in exercise‐based interventions, which inevitably introduced performance bias, as well as inadequate reporting of allocation concealment and outcome assessment blinding in several trials.

### 3.4 Meta‐Analysis Results

#### 3.4.1 Depression Rating Scale Scores

All eight included studies compared the depression rating scale scores of the intervention groups and the control groups (*n* = 487). Among them, the data from Han 2024 [33] only provided median and quartiles, and the mean and standard deviation were obtained after contacting the authors. Because different depression rating scales were used across studies, the standardised mean difference (SMD) was employed as the effect measure. The meta‐analysis showed that the depression rating scale scores in the intervention groups were significantly lower than those in the control groups (SMD = −1.39, 95% CI [−2.22, −0.57], *p* = 0.001), with substantial heterogeneity across studies (τ^2^ = 1.30, χ^2^ = 105.74, df = 7, *p* < 0.00001, I^2^ = 93%). The 95% prediction interval for the true effect in a future study ranged from −4.99 to 2.20. The forest plot of the effectiveness of depression rating scale scores is shown in Figure 5.

**FIGURE 5:**
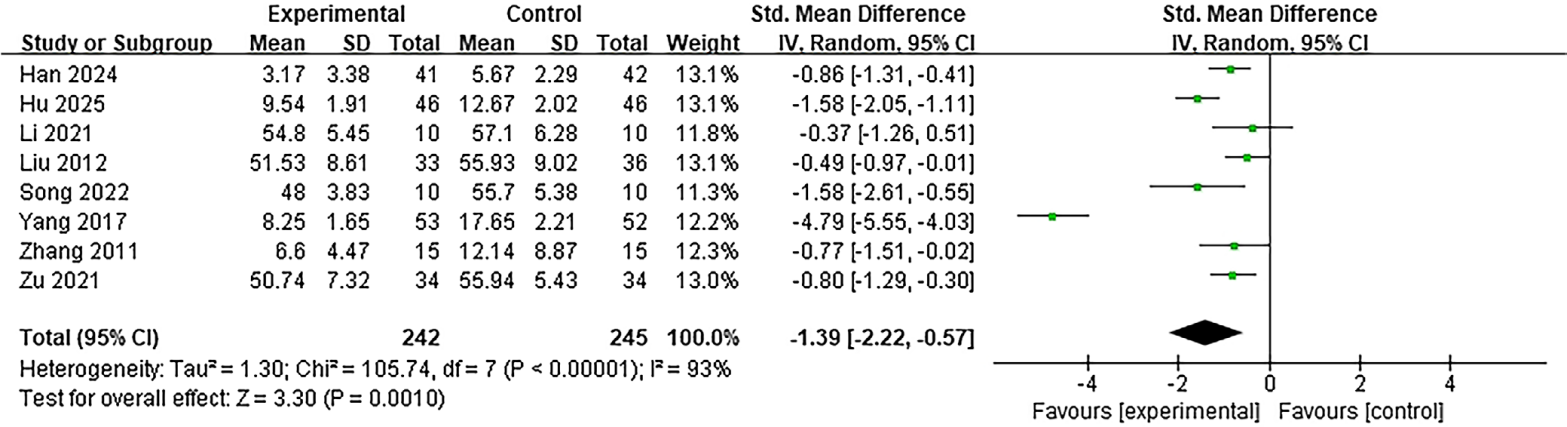
Effectiveness of TCFMs on depression rating scale scores.

#### 3.4.2 HbA1c

Seven studies compared the HbA1c of the intervention groups and the control groups (*n* = 419). The meta‐analysis showed that HbA1c levels in the intervention groups were significantly lower than those in the control groups (MD = −0.82, 95% CI [−1.18, −0.45], *p* < 0.0001), with moderate heterogeneity across studies (τ^2^ = 0.13, *χ* ^2^ = 13.48, df = 6, *p* = 0.04, I^2^ = 55%). The 95% prediction interval for the true effect in a future study ranged from −0.93 to −0.28. The forest plot of the effectiveness of HbA1c is shown in Figure 6.

**FIGURE 6:**
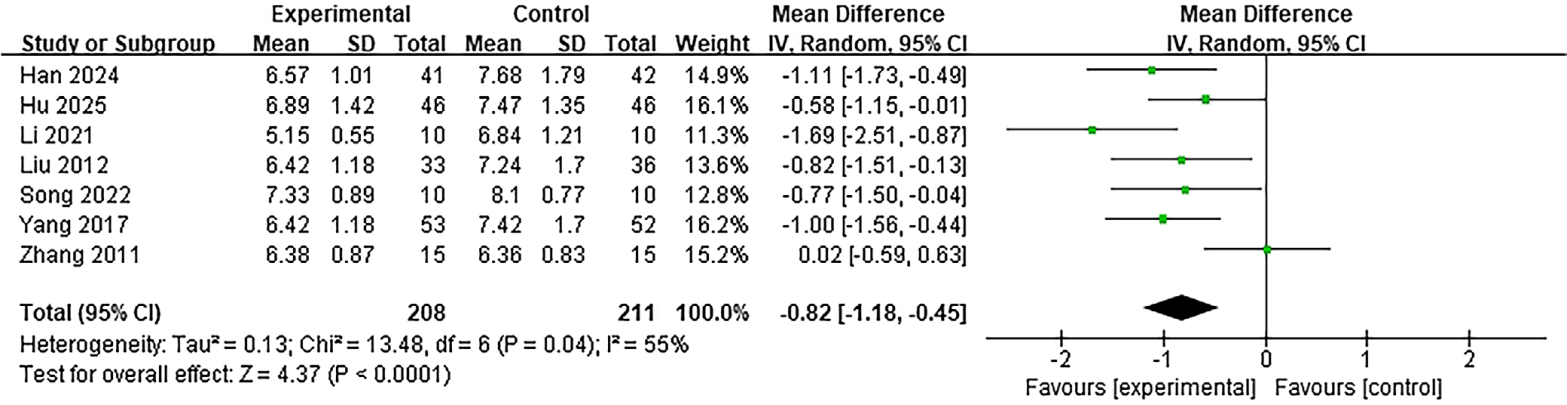
Effectiveness of TCFMs on HbA1c.

#### 3.4.3 FPG

Six studies compared the FPG (mmol/L) of the intervention groups and the control groups (*n* = 313). The meta‐analysis showed that FPG levels in the intervention groups were significantly lower than those in the control groups (MD = −0.70, 95% CI [−0.97, −0.42], *p* < 0.00001), with no heterogeneity across studies (τ^2^ = 0.00, *χ* ^2^ = 4.99, df = 5, *p* = 0.42, I^2^ = 0%). The 95% prediction interval for the true effect in a future study ranged from −0.79 to −0.36. The forest plot of the effectiveness of FPG is shown in Figure 7.

**FIGURE 7:**
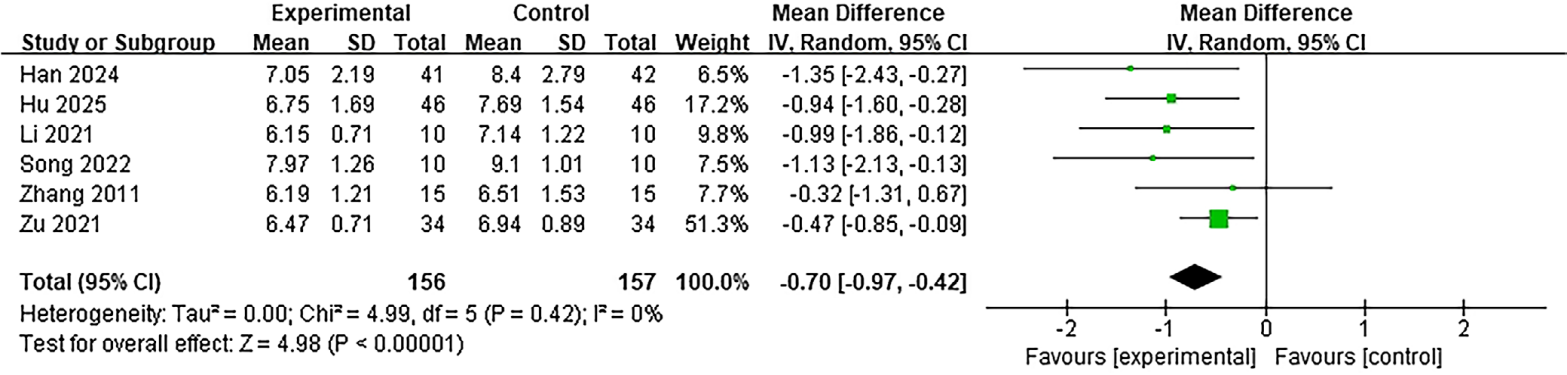
Effectiveness of TCFMs on FPG.

#### 3.4.4 2hPBG

Two studies reported 2hPBG data (*n* = 122) and were therefore excluded from quantitative synthesis due to the prespecified criterion requiring at least three studies for meta‐analysis. Descriptive review of the two trials revealed considerable heterogeneity in intervention type (Baduanjin vs. 24‐form Taijiquan), duration (6 months vs. 8 weeks), intensity, comparator interventions, and effect direction—one study reported a significant between‐group difference in favour of TCFMs, while the other did not.

### 3.5 Subgroup Analyses

Subgroup analyses were performed to explore potential sources of heterogeneity for depression rating scale scores (I^2^ = 93%) and HbA1c (I^2^ = 55%). For both outcomes, subgroup classifications included intervention duration (> 3 vs. ≤ 3 months), frequency (> 5 vs. ≤ 5 days/week), and sample size (> 60 vs. ≤ 60 participants). Other potential effect modifiers (session length, intervention type, combined interventions, age, and baseline depression severity) were not examined owing to insufficient studies or limited baseline variability. All interaction *p*‐values are reported in Tables 3 and 4.

**TABLE 3:** Subgroup Analysis of the score of the depression rating scale.

Variable characteristic	Subgroup	Number of studies	SMD	95% CI	*P*	*I* ^2^ (%)	Interaction *P*
Intervention duration	> 3 months	3	−0.76	−1.12 ~ −0.41	< 0.0001	0	0.12
	≤ 3 months	5	−1.83	−3.11 ~ −0.54	0.005	96	
Intervention frequency	> 5 days/week	5	−1.66	−3.24 ~ −0.09	0.04	96	0.43
	≤ 5 days/week	3	−0.98	−1.59 ~ −0.36	0.002	81	
Sample size	> 60	5	−1.67	−2.82 ~ −0.53	0.004	96	0.22
	≤ 60	3	−0.85	−1.48 ~ −0.23	0.008	35	

**TABLE 4:** Subgroup Analysis of HbA1c.

Variable characteristic	Subgroup	Number of studies	MD	95% CI	*P*	*I* ^2^ (%)	Interaction *P*
Intervention duration	> 3 months	3	−0.90	−1.88 ~ 0.08	0.07	84	0.84
	≤ 3 months	4	−0.79	−1.11 ~ −0.48	< 0.00001	0	
Intervention frequency	> 5 days/week	4	−0.83	−1.50 ~ −0.16	0.01	75	0.98
	≤ 5 days/week	3	−0.82	−1.18 ~ −0.46	< 0.00001	0	
Sample size	> 60	4	−0.87	−1.17 ~ −0.57	< 0.00001	0	0.86
	≤ 60	3	−0.78	−1.75 ~ 0.18	0.11	82	

#### 3.5.1 Depression Rating Scale Scores

Subgroup analyses for depression rating scale scores were performed based on intervention duration, frequency, and sample size, with each subgroup containing at least three studies.

For intervention duration dichotomized at 3 months, the longer‐duration subgroup (> 3 months) showed a smaller effect but with high consistency (SMD = −0.76, 95% CI [−1.12, −0.41], *p* < 0.0001, I^2^ = 0%, 3 studies). In contrast, the shorter‐duration subgroup (≤ 3 months) showed a larger effect but with high variability (SMD = −1.83, 95% CI [−3.11, −0.54], *p* = 0.005, I^2^ = 96%, 5 studies). The between‐subgroup difference was not statistically significant (*p* = 0.12).

For intervention frequency, the pooled effect was statistically significant in both subgroups: > 5 days/week (SMD = −1.66, 95% CI [−3.24, −0.09], *p* = 0.04, I^2^ = 96%, 5 studies) and ≤ 5 days/week (SMD = −0.98, 95% CI [−1.59, −0.36], *p* = 0.002, I^2^ = 81%, 3 studies). The subgroup difference was not statistically significant (*p* = 0.43).

For sample size, the pooled effect was statistically significant in both subgroups: > 60 participants (SMD = −1.67, 95% CI [−2.82, −0.53], *p* = 0.004, I^2^ = 96%, 5 studies) and ≤ 60 participants (SMD = −0.85, 95% CI [−1.48, −0.23], *p* = 0.008, I^2^ = 35%, 3 studies). The subgroup difference was not statistically significant (*p* = 0.22).

Overall, none of the examined variables independently explained the substantial heterogeneity in depressive symptoms, although the marked heterogeneity reduction in the longer‐duration subgroup (I^2^ = 0%) warrants attention. Detailed subgroup results were presented in Table 3.

#### 3.5.2 HbA1c

Subgroup analyses for HbA1c were performed for intervention duration (> 3 vs. ≤ 3 months), intervention frequency (> 5 vs. ≤ 5 days/week), and sample size (> 60 vs. ≤ 60 participants), as these variables had sufficient studies in each subgroup (at least three studies) to support reliable comparisons.

For intervention duration, studies with > 3 months showed a non‐significant trend (MD = −0.90, 95% CI [−1.88, 0.08], *p* = 0.07, I^2^ = 84%, 3 studies), while studies with ≤ 3 months showed a significant effect (MD = −0.79, 95% CI [−1.11, −0.48], *p* < 0.00001, I^2^ = 0%, 4 studies). The subgroup difference was not statistically significant (*p* = 0.84).

For intervention frequency, the pooled effect was statistically significant in both subgroups: > 5 days/week (MD = −0.83, 95% CI [−1.50, −0.16], *p* = 0.01, I^2^ = 75%, 4 studies) and ≤ 5 days/week (MD = −0.82, 95% CI [−1.18, −0.46], *p* < 0.00001, I^2^ = 0%, 3 studies). The subgroup difference was not statistically significant (*p* = 0.98).

For sample size, the pooled effect was statistically significant in the > 60 participants subgroup (MD = −0.87, 95% CI [−1.17, −0.57], *p* < 0.00001, I^2^ = 0%, 4 studies), while the ≤ 60 participants subgroup showed a non‐significant trend (MD = −0.78, 95% CI [−1.75, 0.18], *p* = 0.11, I^2^ = 82%, 3 studies). The subgroup difference was not statistically significant (*p* = 0.86).

Overall, none of the examined variables significantly modified the effect of TCFMs on HbA1c, suggesting that the moderate heterogeneity (I^2^ = 55%) observed in the overall analysis was not attributable to these variables. Detailed subgroup results are presented in Table 4.

Detailed forest plots for all subgroup analyses are provided in Figures S1–, S6.

### 3.6 Meta‐Regression Analysis

Meta‐regression was not performed because the number of included studies was fewer than 10 (*n* = 8), which is below the recommended threshold for reliable meta‐regression analysis as specified in the protocol.

### 3.7 Sensitivity Analysis

Sensitivity analyses were performed using the leave‐one‐out approach to assess the robustness of the pooled effect estimates. For the primary outcome (depression rating scale scores), additional sequential exclusions were conducted for studies with unclear randomisation [30], master's theses or dissertations [27, 30, 32], high risk of bias [27, 28, 29, 30, 31, 34], combined interventions [34], and extreme effect sizes [29]. Across all sensitivity analyses, the pooled SMDs ranged from −0.91 to −1.67, and all remained statistically significant and directionally consistent (all *p* < 0.01), supporting the robustness of the findings for depressive symptoms. Notably, when studies at high risk of bias were excluded, heterogeneity decreased markedly from 93% to 36%, suggesting that methodological quality was a substantial contributor to the observed heterogeneity.

To assess the robustness of the primary finding to the choice of statistical method, we re‐analysed the depression outcome using the HKSJ method. The HKSJ analysis yielded a point estimate similar to the DerSimonian‐Laird method (HKSJ: SMD = −1.35, 95% CI [−2.41, −0.29], *p* = 0.03 vs. DL: SMD = −1.39, 95% CI [−2.22, −0.57], *p* = 0.001), with the confidence intervals being wider as expected with the HKSJ approach. The direction, magnitude, and statistical significance of the pooled effect were consistent across both methods, confirming that the findings for the primary outcome are robust to the choice of statistical model. The forest plot of the HKSJ sensitivity analysis is provided in Figure S7.

For HbA1c and FPG, the exclusion of individual studies did not meaningfully alter the conclusions. For 2hPBG, which was not pooled due to the insufficient number of studies, the descriptive sensitivity check indicated that the results were highly sensitive to the inclusion of either trial; therefore, this outcome was excluded from the quantitative synthesis.

### 3.8 Publication Bias Analysis

Formal publication bias assessment using funnel plots was not conducted, as the number of studies for the primary outcome was fewer than 10 (*n* = 8). However, several clinically oriented indicators suggest that publication bias may be present. All eight included studies reported positive findings favouring TCFMs, which is unlikely to occur by chance if truly null or negative results were equally publishable. Moreover, all studies were conducted in China, and most had small sample sizes. These characteristics are consistent with a high risk of publication bias. For secondary outcomes (HbA1c, FPG, and 2hPBG), the number of available studies was even smaller (ranging from 2 to 7), precluding any formal assessment of publication bias.

### 3.9 Quality of Evidence

The quality of evidence for each outcome was assessed using the GRADE approach, as presented in Table 5. For the depression rating scale score, the certainty of evidence was rated as low, downgraded by one level for serious risk of bias (measurement and implementation bias) and by another level for unexplained high heterogeneity across studies. For HbA1c and FPG, the certainty was rated as moderate, each downgraded by one level solely for risk of bias. For 2hPBG, the certainty was rated as very low, downgraded for risk of bias, serious inconsistency (due to inconsistent direction of effects across the two studies), and indirectness due to the limited evidence base. Overall, the certainty of evidence across the included outcomes ranged from moderate to very low.

**TABLE 5:** Summary of Findings for the GRADE assessment.

Outcome	Effect size (95% CI)	No. of participants (studies)	Quality of evidence	Downgrade factors
Depression rating scale score	SMD = −1.37 (−2.35, −0.38)	395 (8)	Low ⊕ ⊕ ⊝⊝	①②
HbA1c	MD = −0.82 (−1.18, −0.45)	419 (7)	Moderate ⊕ ⊕ ⊕⊝	①
FPG	MD = −0.70 (−0.97, −0.42)	313 (6)	Moderate ⊕ ⊕ ⊕⊝	①
2hPBG	MD = −0.77 (−2.66, 1.12)	122 (2)	Very low ⊕⊝⊝⊝	②③

*Note:* Downgrade Factor Explanations: ① Significant risks of measurement bias and implementation bias exist in the included studies. ② Hierarchical subgroup variables fail to explain the source of overall high heterogeneity. ③ The pooled confidence interval crosses the invalid line bidirectionally, with a severely insufficient total sample size. GRADE Evidence Quality Definition: ⊕ ⊕ ⊕ ⊕ High quality: Further research is very unlikely to change our confidence in the effect estimate. ⊕ ⊕ ⊕ ⊝ Moderate quality: Further research may have an important impact on our confidence in the effect estimate and may change the estimate. ⊕ ⊕⊝⊝ Low quality: Further research is very likely to have an important impact on our confidence in the effect estimate and is likely to change the estimate. ⊕ ⊝⊝⊝ Very low quality: We have very limited confidence in the effect estimate.

Abbreviations: CI, Confidence Interval; FPG, Fasting Plasma Glucose; 2hPBG, 2‐h Postprandial Blood Glucose; HbA1c, Glycated Haemoglobin; MD, Mean Difference; SMD, Standardised Mean Difference.

## 4 Discussion

This systematic review and meta‐analysis suggests that TCFMs may alleviate depressive symptoms and improve glycaemic control (HbA1c and FPG) in patients with T2DM and comorbid depression, while the effect on 2hPBG remains inconclusive due to the limited number of available studies. Eight RCTs were included, most of which clearly presented the randomisation method and fully reported outcomes. The TCFMs examined were predominantly Taijiquan and Baduanjin—exercises that are relatively easy to learn, widely applied, and well‐represented in the literature [35]. Intervention protocols varied, with session length typically ranging from 30 to 60 min, frequency from 3 to 7 sessions per week, and total duration from 1 to 6 months. Collectively, the pooled findings suggest that TCFMs hold promise as an adjunctive intervention for this population. However, this promise is tempered by substantial statistical heterogeneity, methodological shortcomings, and the geographic restriction of the included trials to China. Six of the eight included studies were judged to have a high overall risk of bias, and none was classified as having a low overall risk, raising the possibility that the pooled effects may be exaggerated. Consequently, the current evidence should be considered preliminary and hypothesis‐generating, and the observed effect estimates should not be interpreted as definitive or directly translatable to clinical practice.

In traditional Chinese medicine (TCM), diabetes is historically associated with emotional disturbances, including depression [36, 37]. Taijiquan integrates martial‐arts elements with meditative practice; through slow, continuous, dance‐like movement sequences, it promotes mind–body balance and rehabilitation [38]. Baduanjin, a traditional Daoyin Qigong form, consists of eight extended and graceful movements and emphasises coordinated regulation of posture, breath, and intention, which are traditionally understood to harmonise qi and blood and to loosen muscles and joints [39]. From a TCM perspective, these benefits are theoretically attributed to Qi regulation, Yin‐Yang balance, and liver‐soothing [36, 37]. While these traditional concepts provide a culturally grounded framework, they remain largely hypothesis‐generating and await rigorous mechanistic validation. From a biomedical perspective, these characteristics may reduce depressive symptoms through experimentally supported pathways, including dopamine/endorphin release, cortisol reduction, and improved metabolic function [13, 14, 15], consistent with systematic review evidence on mind–body exercises [40]. Regarding glycaemic outcomes, gentle rhythmic muscle contractions enhance circulation, modulate insulin secretion, and improve insulin sensitivity [41], while intentional breath‐movement coordination influences hypothalamic energy metabolism and reduces sympathetic activation and catecholamine release [42, 43]. Beyond these pathways, TCFMs may also engage mechanisms distinct from general physical activity by integrating slow movements with meditative attention and breath regulation. These mind–body components may enhance vagal tone, modulate autonomic balance, attenuate hypothalamic–pituitary–adrenal (HPA) axis activity, and reduce systemic inflammation—a pathway implicated in both insulin resistance and depression—thereby contributing to glycaemic control and mood regulation [40, 41, 42]. However, the additional effects of these mind–body components beyond general physical activity remain unclear.

Considerable variation in intervention frequency and duration was observed across the included trials. Given that patients with T2DM and comorbid depression often exhibit suboptimal adherence to exercise and self‐management [44], exploring whether these intervention characteristics influence treatment outcomes is of considerable clinical importance. Accordingly, subgroup analyses were performed by intervention duration, frequency, and sample size. However, none of the examined variables significantly modified the treatment effect for either depressive symptoms or HbA1c (all interaction *p* > 0.05). The consistently null findings may be attributable to the limited number of studies available for each subgroup comparison, which reduces statistical power and precludes reliable detection of true effect modifications. Additionally, substantial heterogeneity persisted within most subgroups, suggesting that unmeasured methodological and clinical differences across studies may have obscured potential subgroup effects. Consequently, these subgroup findings should be interpreted as exploratory and hypothesis‐generating rather than as providing definitive evidence against the presence of effect modifiers.

The substantial statistical heterogeneity observed in the primary outcome indicates that the pooled effect estimate may not represent a single true effect applicable to all populations and interventions. Rather, the true effects of TCFMs on depressive symptoms are likely to vary considerably across different intervention protocols, participant characteristics, and study settings. This is further supported by the wide prediction interval for depressive symptoms (Figure 5), which crossed the null and suggests that in some future settings the effect may not be clinically meaningful. The HKSJ‐based prediction interval was similarly wide and crossed the null (Figure S7), consistent with this interpretation. The persistently high heterogeneity (I^2^ ≥ 50%) observed across subgroup analyses suggests that methodological and clinical variations among studies may have obscured the true intervention effects. Sources of this variability included differences in intervention protocol standardisation, baseline participant characteristics, and outcome measurement instruments. Beyond these, other potential effect modifiers—such as intervention type, the specific depression rating scale used, and baseline depression severity—could not be formally examined owing to the limited number of studies in each category or restricted baseline variability, precluding meaningful stratification. Consequently, the contribution of these untested factors to the observed heterogeneity remains unknown.

Sensitivity analyses confirmed the robustness of the primary findings for depressive symptoms, with the pooled effect estimates remaining statistically significant across all prespecified exclusions. Notably, when studies at high risk of bias were excluded, heterogeneity decreased markedly from 93% to 36%, suggesting that methodological quality was a major contributor to the observed heterogeneity. However, the fact that sensitivity analyses remained statistically significant does not eliminate the possibility that consistently biased studies can still produce statistically stable but unreliable pooled estimates. Of particular note, one study (Yang 2017 [29]) showed an exceptionally large effect size and was identified as an extreme outlier. Although its intervention frequency and baseline depression severity were comparable to other included trials, the reason for this extreme effect remains unclear, though it may be related to differences in the study population, implementation of the intervention, or unmeasured confounding factors. Exclusion of this outlier substantially reduced the pooled SMD and heterogeneity, suggesting that the extreme effect size inflated the overall estimate and contributed considerably to the observed heterogeneity; however, the direction and statistical significance of the pooled effect remained unchanged. For HbA1c and FPG, descriptive sensitivity checks suggested that the pooled estimates were not unduly influenced by any single study, supporting the robustness of the glycaemic findings.

Despite these limitations, the observed effect sizes suggest potential clinical relevance that warrants further investigation. The moderate‐to‐large effect size for depressive symptom improvement suggests TCFMs may offer perceptible benefits for mood regulation, which is clinically relevant as comorbid depression drives poor glycaemic control and reduced quality of life in T2DM patients [45]. For glycaemic outcomes, the HbA1c reduction approached the minimal clinically important difference of approximately 0.5%–1.0% [46] and is expected to translate into decreased microvascular complication risk, based on findings from the UK Prospective Diabetes Study [47]. The improvement in FPG also supports the potential role of TCFMs in comprehensive glycaemic management. However, the effect on 2hPBG remains inconclusive due to limited studies. Collectively, TCFMs may serve as a promising adjunctive approach for managing both psychological and metabolic outcomes, but confirmation in high‐quality trials is required before clinical recommendation. Nevertheless, given the considerable heterogeneity and generally low methodological quality of the included studies, these findings should be interpreted with caution.

Beyond the evidence base itself, this study has several inherent limitations that should be acknowledged. First, none of the included trials systematically reported adverse events or addressed issues of cost and accessibility. This absence does not indicate that TCFMs are safe; rather, it precludes any meaningful assessment of their safety profile. Adherence, acceptability, feasibility, cost, accessibility, and long‐term sustainability were also not reported in any included trial, representing important evidence gaps that future research should address. Second, the restriction to publicly published studies—with no systematic grey literature search—may have introduced publication biases; this concern is reinforced by the observation that all eight included studies reported positive findings. Third, the included studies exhibited generally low methodological quality and small sample sizes, which compromise internal validity, while the geographic restriction to China limits the generalisability of the results. Fourth, only two studies measured 2hPBG, precluding any meaningful quantitative synthesis for this outcome. Given these limitations, the pooled effect estimates should be interpreted with caution, and the findings should be considered preliminary and hypothesis‐generating rather than definitive. Collectively, these limitations indicate that the current evidence base is insufficient to support definitive clinical recommendations, underscoring the promising potential of TCFMs while highlighting the critical need for confirmation through rigorously designed, adequately powered trials.

Future research should prioritise three critical improvements: Standardising intervention protocols across modalities, increasing sample sizes with refined population stratification, and unifying outcome measurement tools to enhance comparability. These steps will help clarify optimal intervention parameters, define applicable patient subgroups, and ultimately improve the certainty and clinical translatability of the evidence. Where feasible, larger sample sizes and multicentre trials are recommended to enhance generalisability. In addition, head‐to‐head comparative trials are needed to determine whether one TCFM modality confers superior benefits over the other, and dose–response trials are required to confirm the observed threshold effects of intervention duration on depressive symptom stability. Finally, future studies should incorporate mechanistic components—such as biomarker assessments or neuroimaging—to clarify whether the mind–body components unique to TCFMs can provide additional clinical benefits beyond general physical exercise.

## 5 Conclusions

This systematic review and meta‐analysis suggests that TCFMs may alleviate depressive symptoms and improve glycaemic control (HbA1c and FPG) in patients with T2DM and comorbid depression, while the effect on 2hPBG remains inconclusive. However, the overall certainty of this evidence is low, primarily due to substantial statistical heterogeneity (I^2^ = 93%), generally low methodological quality, and the geographic restriction of all included trials to China. Subgroup analyses did not identify significant effect modification by intervention duration, frequency, or sample size; however, given the limited number of included studies, this null finding should be interpreted with caution. Sensitivity analyses confirmed the robustness of the primary findings, but the high risk of bias in most included studies limits the confidence in these estimates. Given that these findings are still preliminary, rigorously designed, adequately powered multicentre randomised controlled trials with standardised protocols, blinding, and long‐term follow‐up are needed to confirm these preliminary findings, along with assessments of both efficacy and safety, to provide a more robust evidence base for clinical decisions.

## Author Contributions

**Xinyue Tian:** data curation, validation, formal analysis, writing – review and editing, writing – original draft, investigation, visualization. **Yiwen Wei:** data curation, formal analysis, investigation, writing – original draft, writing – review and editing. **Jingwen Bo:** investigation, formal analysis, data curation, writing – review and editing, writing – original draft. **Keke Lin:** visualization, conceptualization, writing – review and editing, methodology. **Yina Shen:** conceptualization, writing – original draft, validation, writing – review and editing. **Yu Liu:** conceptualization, writing – original draft, methodology, writing – review and editing, supervision.

## Funding

The authors have nothing to report.

## Ethics Statement

Our study did not involve any human or animal subjects; therefore, no IRB (or IACUC) approval was required.

## Consent

Our study did not involve scenarios requiring patient informed consent; therefore, no patient informed consent was required.

## Conflicts of Interest

The authors declare no conflicts of interest.

## Supporting information


**Figure S1:** Forest plot of subgroup analysis for depression rating scale scores by intervention duration.

**Figure S2:** Forest plot of subgroup analysis for depression rating scale scores by intervention frequency.

**Figure S3:** Forest plot of subgroup analysis for depression rating scale scores by sample size.

**Figure S4:** Forest plot of subgroup analysis for HbA1c by intervention duration.

**Figure S5:** Forest plot of subgroup analysis for HbA1c by intervention frequency.

**Figure S6:** Forest plot of subgroup analysis for HbA1c by sample size.

**Figure S7:** Hartung‐Knapp‐Sidik‐Jonkman (HKSJ) random‐effects meta‐analysis of depression rating scale scores (supplementary sensitivity analysis).

**File S1:** PRISMA 2020 checklist.

**Table S1:** Search Strategies.
**Table S2:** Detailed baseline characteristics of the eight included studies (T = intervention group, C = control group; MADRS = Montgomery‐Asberg Depression Rating Scale; SDS = Self‐Rating Depression Scale; HAMD = Hamilton Depression Scale; PHQ‐9 = Patient Health Questionnaire‐9).

## Data Availability

Data are included in the attached documents and are presented as tables and figures. We declare the availability of the data used in this article and ensure the data are transparent and explained when the data cannot be used.
