# Supplementary material for: Effectiveness of Traditional Chinese Fitness Methods on Depressive Symptoms and Glycaemic Control in Patients With Type 2 Diabetes and Comorbid Depression: A Systematic Review and Meta‐Analysis of Taijiquan and Baduanjin
Source: Endocrinol Diabetes Metab. 2026 Nov 1;9(6):e70356. doi: 10.1002/edm2.70356 (PMC13724216; doi:10.1002/edm2.70356)

Study or Subgroup	Experimental Mean	Experimental SD	Experimental Total	Control Mean	Control SD	Control Total	Weight	Std. Mean Difference IV, Random, 95% CI
**1.6.1 Intervention frequency ＞ 5days/week**								
Li 2021	54.8	5.45	10	57.1	6.28	10	11.8%	-0.37 [-1.26, 0.51]
Song 2022	48	3.83	10	55.7	5.38	10	11.3%	-1.58 [-2.61, -0.55]
Yang 2017	8.25	1.65	53	17.65	2.21	52	12.2%	-4.79 [-5.55, -4.03]
Zhang 2011	6.6	4.47	15	12.14	8.87	15	12.3%	-0.77 [-1.51, -0.02]
Zu 2021	50.74	7.32	34	55.94	5.43	34	13.0%	-0.80 [-1.29, -0.30]
**Subtotal (95% CI)**			**122**			**121**	**60.7%**	**-1.66 [-3.24, -0.09]**
Heterogeneity: Tau² = 3.07; Chi² = 89.08, df = 4 (P < 0.00001); I² = 96%								
Test for overall effect: Z = 2.07 (P = 0.04)								
**1.6.2 Intervention frequency ⩽ 5days/week**								
Han 2024	3.17	3.38	41	5.67	2.29	42	13.1%	-0.86 [-1.31, -0.41]
Hu 2025	9.54	1.91	46	12.67	2.02	46	13.1%	-1.58 [-2.05, -1.11]
Liu 2012	51.53	8.61	33	55.93	9.02	36	13.1%	-0.49 [-0.97, -0.01]
**Subtotal (95% CI)**			**120**			**124**	**39.3%**	**-0.98 [-1.59, -0.36]**
Heterogeneity: Tau² = 0.24; Chi² = 10.46, df = 2 (P = 0.005); I² = 81%								
Test for overall effect: Z = 3.11 (P = 0.002)								
**Total (95% CI)**			**242**			**245**	**100.0%**	**-1.39 [-2.22, -0.57]**
Heterogeneity: Tau² = 1.30; Chi² = 105.74, df = 7 (P < 0.00001); I² = 93%								
Test for overall effect: Z = 3.30 (P = 0.0010)								
Test for subgroup differences: Chi² = 0.63, df = 1 (P = 0.43), I² = 0%								

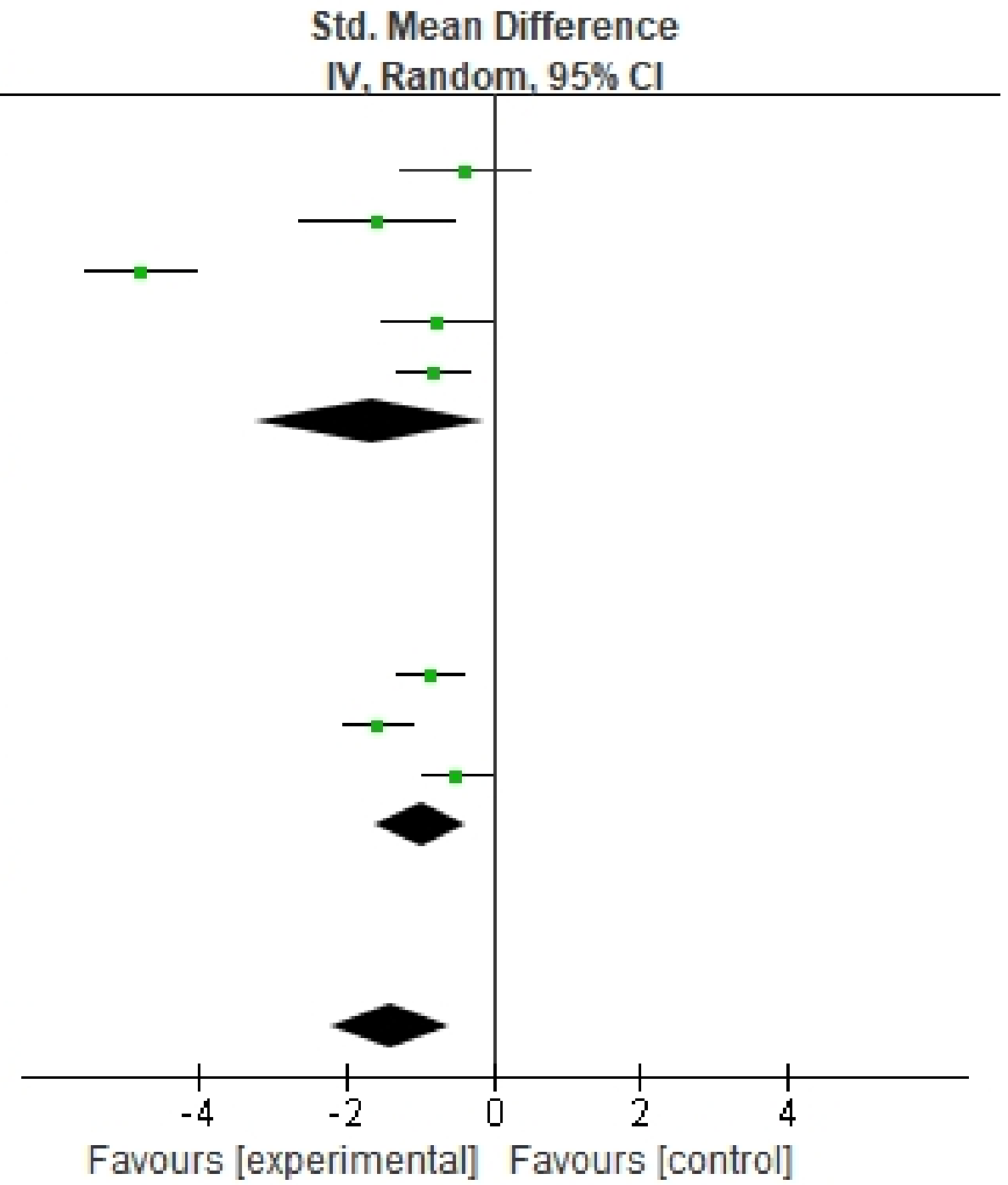

Supplement: Supplementary file 2 [file EDM2-9-e70356-s002.pdf]
