# Supplementary material for: Effectiveness of Traditional Chinese Fitness Methods on Depressive Symptoms and Glycaemic Control in Patients With Type 2 Diabetes and Comorbid Depression: A Systematic Review and Meta‐Analysis of Taijiquan and Baduanjin
Source: Endocrinol Diabetes Metab. 2026 Nov 1;9(6):e70356. doi: 10.1002/edm2.70356 (PMC13724216; doi:10.1002/edm2.70356)

Study or Subgroup	Experimental Mean	Experimental SD	Experimental Total	Control Mean	Control SD	Control Total	Weight	Mean Difference IV, Random, 95% CI
**1.11.1 Intervention duration ＞ 3 moths**								
Han 2024	6.57	1.01	41	7.68	1.79	42	14.9%	-1.11 [-1.73, -0.49]
Li 2021	5.15	0.55	10	6.84	1.21	10	11.3%	-1.69 [-2.51, -0.87]
Zhang 2011	6.38	0.87	15	6.36	0.83	15	15.2%	0.02 [-0.59, 0.63]
**Subtotal (95% CI)**			**66**			**67**	**41.3%**	**-0.90 [-1.88, 0.08]**
Heterogeneity: Tau² = 0.62; Chi² = 12.40, df = 2 (P = 0.002); I² = 84%								
Test for overall effect: Z = 1.81 (P = 0.07)								
**1.11.2 Intervention duration ≤ 3 moths**								
Hu 2025	6.89	1.42	46	7.47	1.35	46	16.1%	-0.58 [-1.15, -0.01]
Liu 2012	6.42	1.18	33	7.24	1.7	36	13.6%	-0.82 [-1.51, -0.13]
Song 2022	7.33	0.89	10	8.1	0.77	10	12.8%	-0.77 [-1.50, -0.04]
Yang 2017	6.42	1.18	53	7.42	1.7	52	16.2%	-1.00 [-1.56, -0.44]
**Subtotal (95% CI)**			**142**			**144**	**58.7%**	**-0.79 [-1.11, -0.48]**
Heterogeneity: Tau² = 0.00; Chi² = 1.08, df = 3 (P = 0.78); I² = 0%								
Test for overall effect: Z = 4.99 (P < 0.00001)								
**Total (95% CI)**			**208**			**211**	**100.0%**	**-0.82 [-1.18, -0.45]**
Heterogeneity: Tau² = 0.13; Chi² = 13.48, df = 6 (P = 0.04); I² = 55%								
Test for overall effect: Z = 4.37 (P < 0.0001)								
Test for subgroup differences: Chi² = 0.04, df = 1 (P = 0.84), I² = 0%								

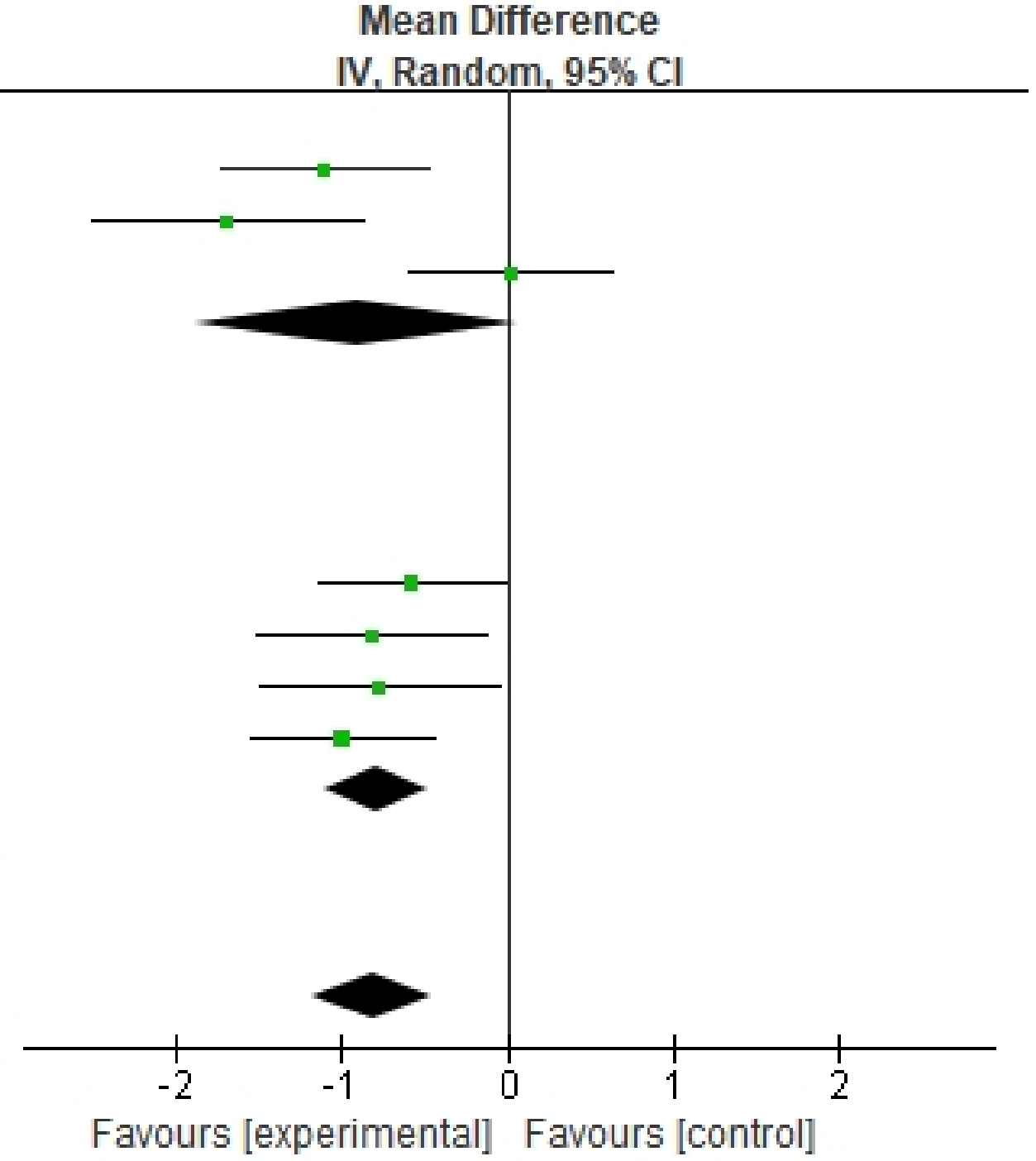

Supplement: Supplementary file 3 [file EDM2-9-e70356-s004.pdf]
