# Supplementary material for: Effectiveness of Traditional Chinese Fitness Methods on Depressive Symptoms and Glycaemic Control in Patients With Type 2 Diabetes and Comorbid Depression: A Systematic Review and Meta‐Analysis of Taijiquan and Baduanjin
Source: Endocrinol Diabetes Metab. 2026 Nov 1;9(6):e70356. doi: 10.1002/edm2.70356 (PMC13724216; doi:10.1002/edm2.70356)
Supplement: Supplementary file 6 [file EDM2-9-e70356-s007.pdf]

Study	Treatment N	Treatment Mean	Treatment SD	Control N	Control Mean	Control SD	Hedges's g with 95% CI	Weight (%)
Han 2024	41	3.17	3.38	42	5.67	2.29	-0.86 [-1.31, -0.41]	12.91
Hu 2025	46	9.54	1.91	46	12.67	2.02	-1.58 [-2.04, -1.11]	12.88
Li 2021	10	54.8	5.45	10	57.1	6.28	-0.37 [-1.22, 0.47]	12.08
Liu 2012	33	51.53	8.61	36	55.93	9.02	-0.49 [-0.97, -0.02]	12.86
Song 2022	10	48	3.83	10	55.7	5.38	-1.58 [-2.55, -0.61]	11.75
Yang 2017	53	8.25	1.65	52	17.65	2.21	-4.79 [-5.54, -4.04]	12.31
Zhang 2011	15	6.6	4.47	15	12.14	8.87	-0.77 [-1.49, -0.04]	12.38
Zu 2021	34	50.74	7.32	34	55.94	5.43	-0.80 [-1.29, -0.31]	12.84
**Overall**							-1.40 [-2.59, -0.20]	

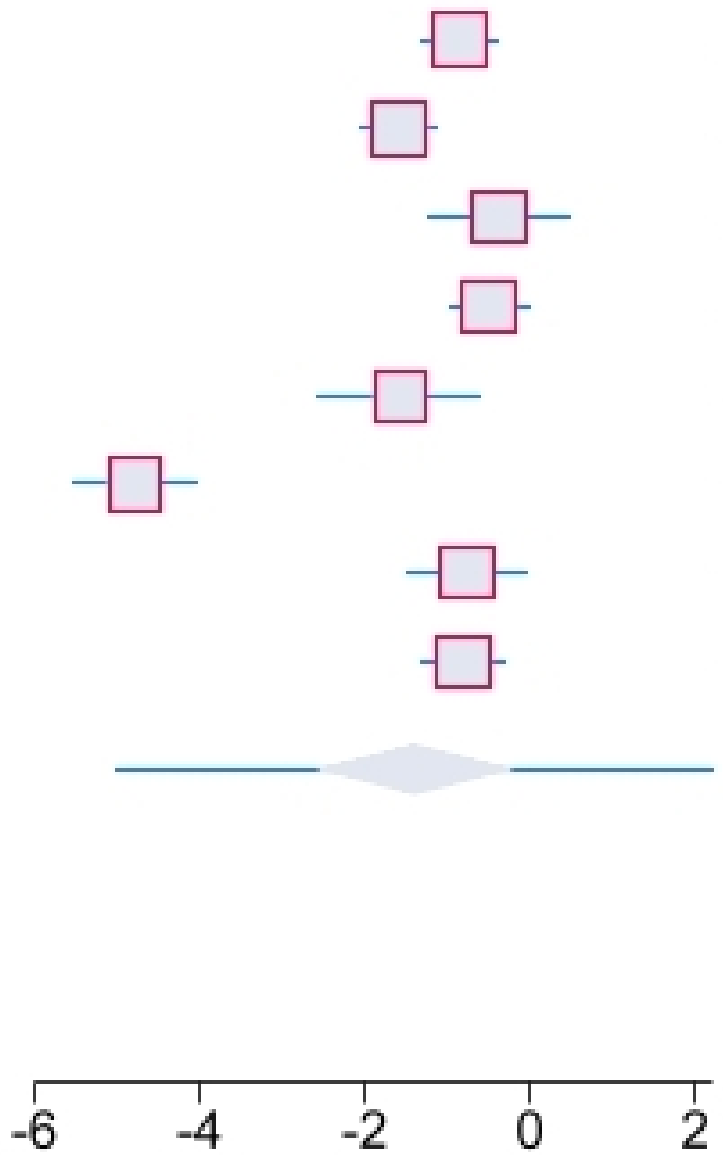

Heterogeneity: $\tau^2 = 1.92$, $I^2 = 95.60\%$, $H^2 = 22.71$

Test of $\theta_i = \theta_j$: Q(7) = 109.36, p = 0.00

Test of $\theta = 0$: t(7) = -2.76, p = 0.03

Random-effects Sidik – Jonkman model
Knapp – Hartung standard errors
95% prediction interval
