# Supplementary material for: Effectiveness of Traditional Chinese Fitness Methods on Depressive Symptoms and Glycaemic Control in Patients With Type 2 Diabetes and Comorbid Depression: A Systematic Review and Meta‐Analysis of Taijiquan and Baduanjin
Source: Endocrinol Diabetes Metab. 2026 Nov 1;9(6):e70356. doi: 10.1002/edm2.70356 (PMC13724216; doi:10.1002/edm2.70356)

Study or Subgroup	Experimental Mean	Experimental SD	Experimental Total	Control Mean	Control SD	Control Total	Weight	Std. Mean Difference IV, Random, 95% CI
**1.9.1 Sample size ＞60**								
Han 2024	3.17	3.38	41	5.67	2.29	42	13.1%	-0.86 [-1.31, -0.41]
Hu 2025	9.54	1.91	46	12.67	2.02	46	13.1%	-1.58 [-2.05, -1.11]
Liu 2012	51.53	8.61	33	55.93	9.02	36	13.1%	-0.49 [-0.97, -0.01]
Yang 2017	8.25	1.65	53	17.65	2.21	52	12.2%	-4.79 [-5.55, -4.03]
Zu 2021	50.74	7.32	34	55.94	5.43	34	13.0%	-0.80 [-1.29, -0.30]
**Subtotal (95% CI)**			**207**			**210**	**64.6%**	**-1.67 [-2.82, -0.53]**
Heterogeneity: Tau² = 1.63; Chi² = 100.16, df = 4 (P < 0.00001); I² = 96%								
Test for overall effect: Z = 2.86 (P = 0.004)								
**1.9.2 Sample size ≤60**								
Li 2021	54.8	5.45	10	57.1	6.28	10	11.8%	-0.37 [-1.26, 0.51]
Song 2022	48	3.83	10	55.7	5.38	10	11.3%	-1.58 [-2.61, -0.55]
Zhang 2011	6.6	4.47	15	12.14	8.87	15	12.3%	-0.77 [-1.51, -0.02]
**Subtotal (95% CI)**			**35**			**35**	**35.4%**	**-0.85 [-1.48, -0.23]**
Heterogeneity: Tau² = 0.11; Chi² = 3.06, df = 2 (P = 0.22); I² = 35%								
Test for overall effect: Z = 2.67 (P = 0.008)								
**Total (95% CI)**			**242**			**245**	**100.0%**	**-1.39 [-2.22, -0.57]**
Heterogeneity: Tau² = 1.30; Chi² = 105.74, df = 7 (P < 0.00001); I² = 93%								
Test for overall effect: Z = 3.30 (P = 0.0010)								
Test for subgroup differences: Chi² = 1.51, df = 1 (P = 0.22), I² = 33.6%								

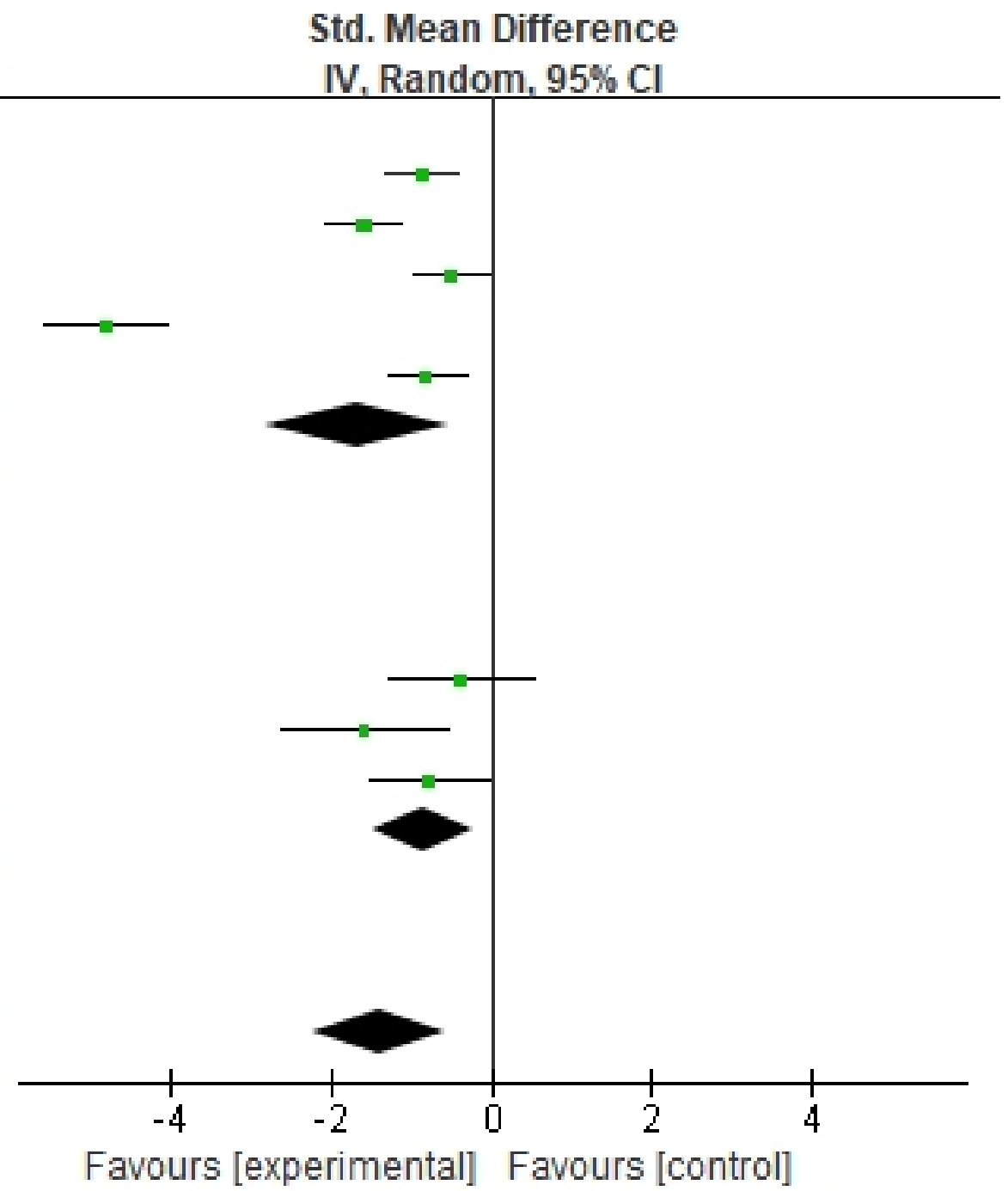

Supplement: Supplementary file 7 [file EDM2-9-e70356-s009.pdf]
