# Supplementary material for: Effectiveness of Traditional Chinese Fitness Methods on Depressive Symptoms and Glycaemic Control in Patients With Type 2 Diabetes and Comorbid Depression: A Systematic Review and Meta‐Analysis of Taijiquan and Baduanjin
Source: Endocrinol Diabetes Metab. 2026 Nov 1;9(6):e70356. doi: 10.1002/edm2.70356 (PMC13724216; doi:10.1002/edm2.70356)
Supplement: Supplementary file 8 [file EDM2-9-e70356-s003.docx]

**Supplementary Table 1.** Search_Strategies

| **Database** | **Search strategies** |
| --- | --- |
| **PubMed** | ("diabetes mellitus"[Mesh] OR diabetes[tiab] OR diabetic[tiab] OR wasting-thirst[tiab]) AND ("depression"[Mesh] OR depressive[tiab]) AND ("Qigong"[Mesh] OR Qigong[tiab] OR chi kung[tiab] OR formula[tiab] OR "Tai Ji"[Mesh] OR taiji[tiab] OR tai chi[tiab] OR T’ai chi[tiab] OR move shadow boxing[tiab] OR ba duan jin[tiab] OR eight section brocade[tiab] OR eight section exercise[tiab] OR five mimic-animal boxing[tiab] OR wuqinxi[tiab] OR five animal exercise[tiab] OR five animal boxing[tiab] OR liuzijue[tiab] OR six character[tiab] OR yijinjing[tiab] OR shierduanjin[tiab] OR twelve section exercise[tiab] OR twelve section brocade[tiab] OR great dance[tiab] OR twelve style daoyin yangsheng gong[tiab] OR guiding health power twelve methods[tiab] OR Mawangdui[tiab] OR psychosomatic relaxation[tiab] OR standing Qigong[tiab]) |
| **EMBASE** | #1 ('diabetes mellitus'/exp OR 'diabetes mellitus') AND [embase]/lim  #2 ('diabetes':ti,ab,kw OR 'diabetic':ti,ab,kw OR 'diabets':ti,ab,kw OR 'unspecified diabetes mellitus':ti,ab,kw OR 'diabetes mellitus':ti,ab,kw)AND [embase]/lim  #3 #1 OR #2  #4 ('depression'/exp OR 'depression') AND [embase]/lim  #5 ('central depression':ti,ab,kw OR 'clinical depression':ti,ab,kw OR 'depressive disease':ti,ab,kw OR 'depressive disorder':ti,ab,kw OR 'depressive episode':ti,ab,kw OR 'depressive illness':ti,ab,kw OR 'depressive personality disorder':ti,ab,kw OR 'depressive state':ti,ab,kw OR 'depressive symptom':ti,ab,kw OR 'depressive syndrome':ti,ab,kw OR 'depressivity':ti,ab,kw OR 'mental depression':ti,ab,kw OR 'parental depression':ti,ab,kw OR 'depression':ti,ab,kw) AND [embase]/lim  #6 #4 OR #5  #7 (qigong:ti,ab,kw OR 'chi kung':ti,ab,kw OR 'standing qigong':ti,ab,kw OR 'psychosomatic relaxation':ti,ab,kw OR formula:ti,ab,kw OR 'tai ji':ti,ab,kw OR 'tai chi':ti,ab,kw OR 'move shadow boxing':ti,ab,kw OR 'ba duan jin':ti,ab,kw OR 'eight section brocade':ti,ab,kw OR 'eight section exercise':ti,ab,kw OR shierduanjin:ti,ab,kw OR 'twelve section brocade':ti,ab,kw OR 'twelve section exercise':ti,ab,kw OR 'five mimic-animal boxing':ti,ab,kw OR wuqinxi:ti,ab,kw OR 'five animal exercise':ti,ab,kw OR 'five animal boxing':ti,ab,kw OR liuzijue:ti,ab,kw OR 'six character':ti,ab,kw OR yijinjing:ti,ab,kw OR mawangdui:ti,ab,kw OR 'twelve style daoyin yangsheng gong':ti,ab,kw OR 'guiding health power twelve methods':ti,ab,kw) AND [embase]/lim  #8 #3 AND #6 AND #7 |
| **Wiley Online**  **Library** | “diabetes mellitus” OR diabetic OR diabetes" in Abstract and "depression OR depressive" in Abstract and "Qigong OR "chi  kung" OR "standing Qigong" OR "psychosomatic relaxation" OR "Tai Ji" OR "Tai Chi" OR "move shadow boxing" OR "ba  duan jin" OR "eight section brocade" OR "eight section exercise" OR shierduanjin OR "twelve section brocade" OR "twelve  section exercise" OR "five mimic-animal boxing" OR wuqinxi OR "five animal exercise" OR "five animal boxing" OR liuzijue OR "six character" OR yijinjing OR Mawangdui OR "twelve style daoyin yangsheng gong" OR "guiding health power twelve methods"" anywhere |
| **CENTRAL** | #1 MeSH descriptor: [Diabetes Mellitus] explode all trees  #2 (diabetic OR diabetes):ti,ab,kw  #3 #1 OR #2  #4 MeSH descriptor: [Depression] explode all trees  #5 (depressive):ti,ab,kw  #6 #4 OR #5  #7 (Qigong OR chi kung OR standing Qigong OR psychosomatic relaxation OR Tai Ji OR Tai Chi OR move shadow boxing  OR ba duan jin OR eight section brocade OR eight section exercise OR shierduanjin OR twelve section brocade OR twelve  section exercise OR five mimic-animal boxing OR wuqinxi OR five animal exercise OR five animal boxing OR liuzijue OR six character OR yijinjing OR Mawangdui OR twelve style daoyin yangsheng gong OR guiding health power twelve  methods):ti,ab,kw  #8 #3 AND #6 AND #7 |
| **Web of**  **Science** | “ diabetes mellitus” OR diabetic OR diabetes (Topic) and depression OR depressive OR “depressive disorder” OR “major depression” (Topic) and Qigong OR "chi kung" OR "standing Qigong" OR "psychosomatic relaxation" OR "Tai Ji" OR "Tai Chi" OR "T’ai chi" OR "move shadow boxing" OR "ba duan jin" OR "eight section brocade" OR "eight section exercise" OR shierduanjin OR "twelve section brocade" OR "twelve section exercise" OR "five mimic-animal boxing" OR wuqinxi OR "five animal exercise" OR "five animal boxing" OR liuzijue OR "six character" OR yijinjing OR Mawangdui OR "twelve style daoyin yangsheng gong" OR "guiding health power twelve methods" (Topic) |
| **ProQuest** | mainsubject("diabetes mellitus" OR diabetic OR diabetes) AND mainsubject(depression OR depressive) AND (Qigong OR "chi kung" OR "standing Qigong" OR "psychosomatic relaxation" OR "Tai Ji" OR "Tai Chi" OR "move shadow boxing" OR "ba  duan jin" OR "eight section brocade" OR "eight section exercise" OR shierduanjin OR "twelve section brocade" OR "twelve  section exercise" OR "five mimic-animal boxing" OR wuqinxi OR "five animal exercise" OR "five animal boxing" OR liuzijue OR "six character" OR yijinjing OR Mawangdui OR "twelve style daoyin yangsheng gong" OR "guiding health power twelve methods") |
| **CBM** | ("糖尿病"[摘要] OR " 消渴"[摘要]) AND ("抑郁"[摘要]) AND ("气功"[摘要] OR "太极" [摘要] OR " 大舞" [摘要] OR " 八段锦" [摘要] OR " 五禽戏" [摘要] OR " 易筋经"[摘要] OR "六字诀"[摘要] OR "静松功"[摘要] OR "放松功"[摘要] OR "站桩功"[摘要] OR "十二段锦"[摘要] OR "马王堆导引术"[摘要] OR "十二式导引养生功"[摘要]) |
| **中国知网** | TKA="糖尿病" + "消渴" AND TKA="抑郁"AND TKA="气功" + "太极" + "大舞" + "八段锦" + "五禽戏" + "易筋经" + "六字诀" + "静松功" + “放松功” + "站桩功" + "十二段锦" + " 马王堆导引术" + "十二式导引养生功" |
| **万方** | 摘要:( "糖尿病" OR "消渴") AND 摘要:("抑郁" ) AND 摘要:("气功" OR "太极" OR " 大舞" OR " 八段锦" OR " 五禽戏" OR " 易筋经" OR " 六字诀" OR "静松功" OR "放松功" OR "站桩功" OR "十二段锦" OR " 马王堆导引术" OR "十二式导引养生功") |
| **维普** | (R=" 糖尿病" OR R=" 消渴") AND (R=" 抑郁") AND (R=" 气功" OR R=" 太极" OR R=" 大舞" OR R=" 八段锦" OR R=" 五禽戏" OR R=" 易筋经" OR R=" 六字诀" OR R="静松功" OR R= “放松功” OR R=" 站桩功" OR R=" 十二段锦" OR R="马王堆导引术" OR R= “十二式导引养生功”) |

**Supplementary Table 2.** Detailed baseline characteristics of the eight included studies

| Research | Sample size(*n*)  T/C | Gender  M/F, T/C | Age (years)  T/C | Diabetes duration (years)  T/C | Baseline HbA1c (%)  T/C | Baseline depression score  T/C, scale | Antidepressant use  T/C | Hypoglycemic drugs | Attrition rate  (%) | Adherence rate |
| --- | --- | --- | --- | --- | --- | --- | --- | --- | --- | --- |
| Zhang 2011 | 15/15 | 6/9,  7/8 | 54.90  (overall) | Not reported | 7.12 ± 1.36 / 7.31 ± 1.71 | 11.67 ± 5.89 / 13.50 ± 9.97 (MADRS) | No / No | Conventional treatment | 16.6 | Not reported |
| Liu 2012 | 33/36 | 14/19, 14/22 | 64.20 ± 7.41 (overall) | 7.58 ± 5.92  (overall) | 7.31 ± 1.48 / 7.41 ± 1.46 | 58.41 ± 4.16 / 58.78 ± 5.13  (SDS) | No / No | Conventional treatment | 21.6 | Not reported |
| Yang 2017 | 53/52 | 26/27, 25/27 | 52.40 ± 2.30 (overall) | 11.20 ± 2.10 / 10.80 ± 1.90 | 7.31 ± 1.48 / 7.41 ± 1.46 | 21.32 ± 2.59 / 21.10 ± 2.69 (HAMD‑24) | No / No | Conventional treatment | 4.5 | Not reported |
| Li 2021 | 10/10 | 5/5,  4/6 | 46.20 ± 7.50 / 44.90 ± 7.49 | 5.15 ± 3.04 /  6.40 ± 4.69 | 6.47 ± 1.05 / 6.89 ± 1.21 | 61.30 ± 5.27 / 58.90 ± 7.56  (SDS) | No / No | Conventional drug therapy | 0 | Not reported |
| Zu 2021 | 34/34 | 19/15, 18/16 | 55.65 ± 10.90 / 55.80 ± 12.30 | 9.30 ± 9.60 /  8.80 ± 9.70 | Not reported | 57.50 ± 5.60 / 57.15 ± 5.53  (SDS) | No / No | Oral hypoglycemics ± insulin | 0 | Not reported |
| Song 2022 | 10/10 | 4/6,  4/6 | 65.70 ± 4.25 / 65.50 ± 4.43 | Not reported | 7.71 ± 1.04 / 8.05 ± 0.76 | 55.70 ± 3.95 / 55.30 ± 5.50  (SDS) | No / No | Conventional drug therapy | 0 | Not reported |
| Han 2024 | 41/42 | 17/24, 18/24 | 65.20 ± 3.52 / 65.05 ± 2.99 | < 10 years: 21/20; ≥ 10 years: 20/26 | 7.06 ± 1.48 / 7.45 ± 1.63 | 7.20 ± 2.91 / 6.52 ± 1.77  (PHQ-9) | No / No | Routine diabetes management | 0 | Not reported |
| Hu 2025 | 46/46 | 24/22, 23/23 | 56.05 ± 6.02 / 55.65 ± 5.93 | 5.98 ± 2.91 /  6.12 ± 2.63 | 8.09 ± 1.61 / 8.13 ± 1.57 | 19.20 ± 2.47 / 18.91 ± 2.62 (HAMD‑17) | Yes (Shugan Jieyu) / Yes (Shugan Jieyu) | Conventional hypoglycemic therapy | 0 | Not reported |
| T = intervention group, C = control group;  MADRS = Montgomery-Asberg Depression Rating Scale; SDS = Self‑Rating Depression Scale;  HAMD = Hamilton Depression Scale; PHQ‑9 = Patient Health Questionnaire‑9. | | | | | | | | | | |
